# Supplementary material for: The apicoplast link to fever-survival and artemisinin-resistance in the malaria parasite
Source: Nat Commun. 2021 Jul 27;12:4563. doi: 10.1038/s41467-021-24814-1 (PMC8316339; doi:10.1038/s41467-021-24814-1)
Supplement: Supplementary file 1 — Supplementary Information [file 41467_2021_24814_MOESM1_ESM.pdf]

The apicoplast link to fever-survival and artemisinin-resistance in the malaria parasite

Zhang M, Wang C, Oberstaller J et al.

SUPPLEMENTARY INFORMATION

Supplementary Figure 1.
Schematic overview of pooled phenotypic screening pipeline.

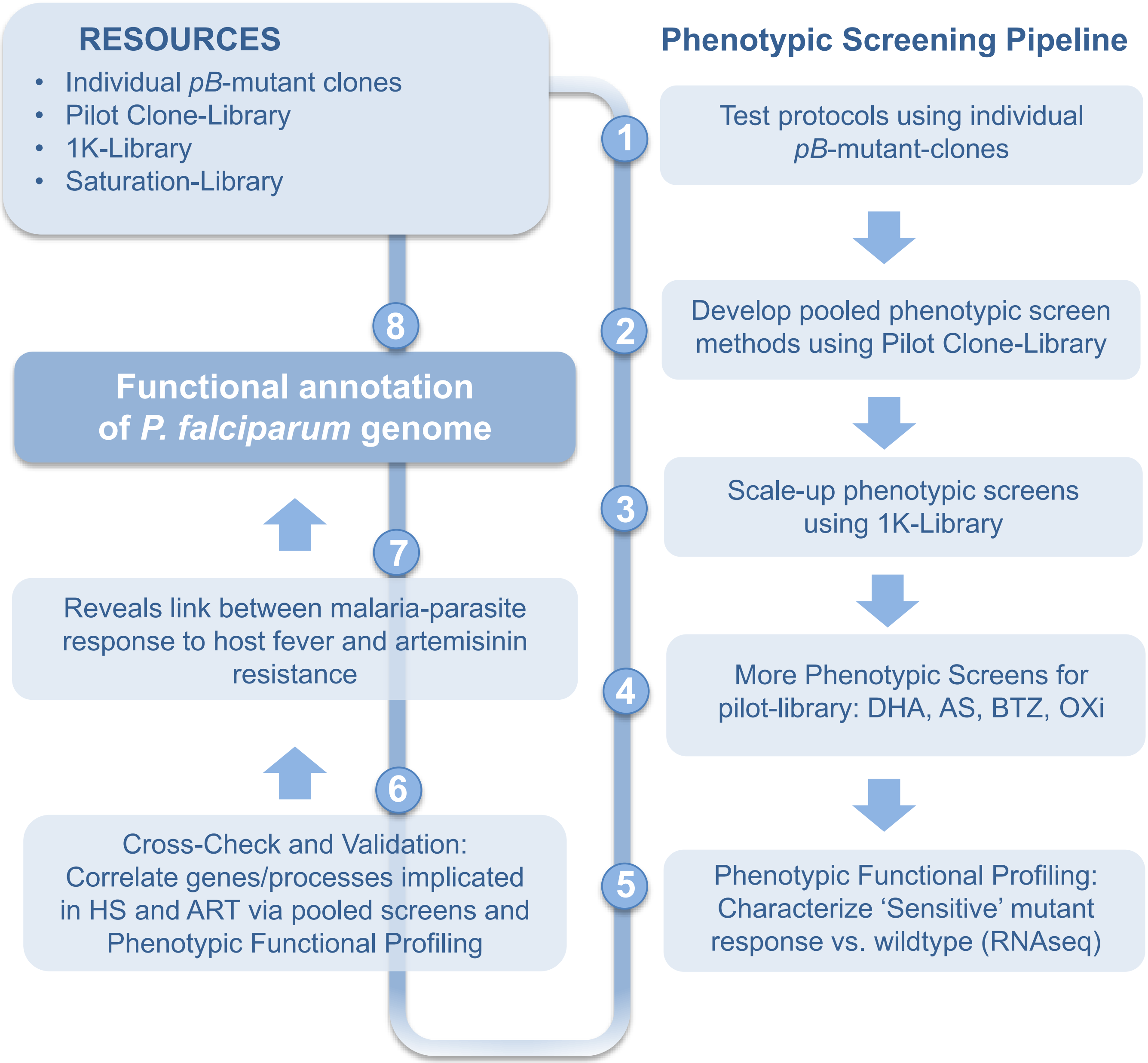

Supplementary Figure 1. Schematic overview of the phenotypic screening pipeline. *pB*-mutant library resources from small (individual, well-characterized mutant-clones) to large (the 1K-Library, comprised of pools randomly selected from the Saturation-Library) were used to design carefully validated pooled screens at increasingly large scale. 1) to test the protocols using individual *pB*-mutant clones; 2) to develop pooled phenotypic screen method using pilot-library screen; 3) then we scale-up phenotypic screen using 1K-library; 4) parallel phenotype screens using pilot-library; 5) Transcriptional profiling via RNAseq compare the parasite response to heat shock between the wildtype and HS-sensitive mutants. High correlation between mutant-phenotypes in HS-screens and ART-screens indicated mechanistic overlap in response to both stressors. Iterative rounds of pooled-screening for various phenotypes over time enables higher-throughput functional-annotation of the *P. falciparum* genome.

Supplementary Figure 2.
Extended screening data against the pilot-library and summary.

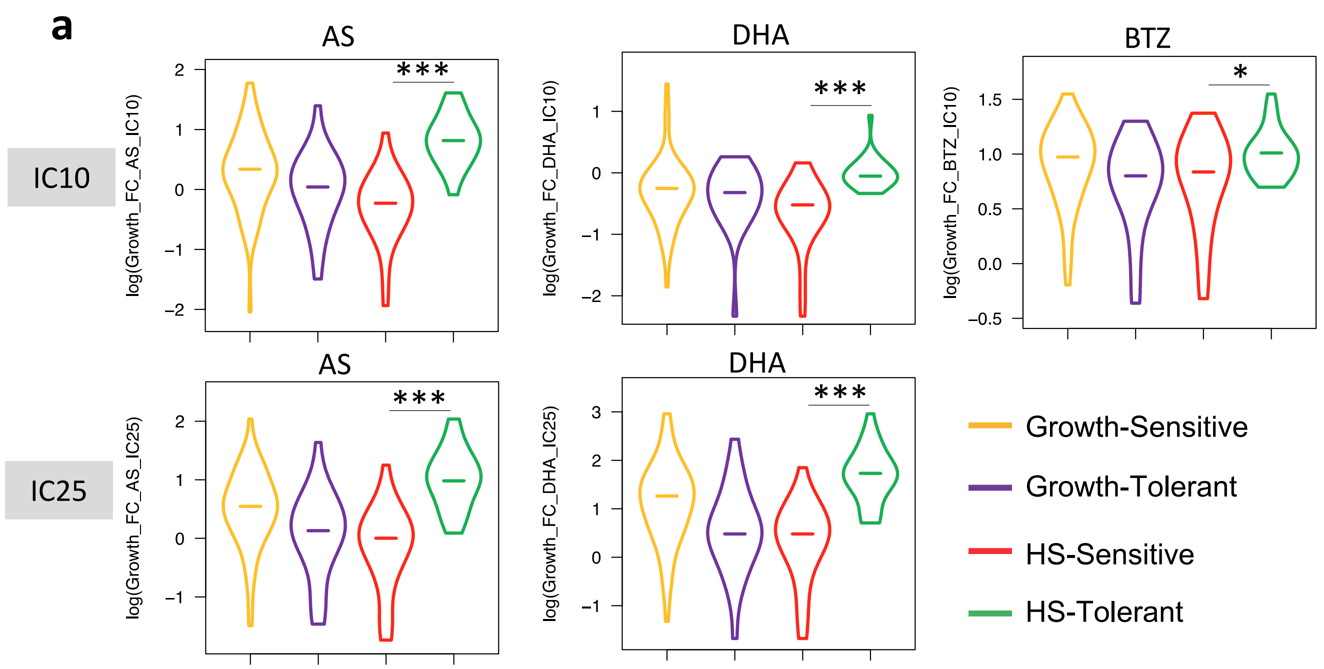

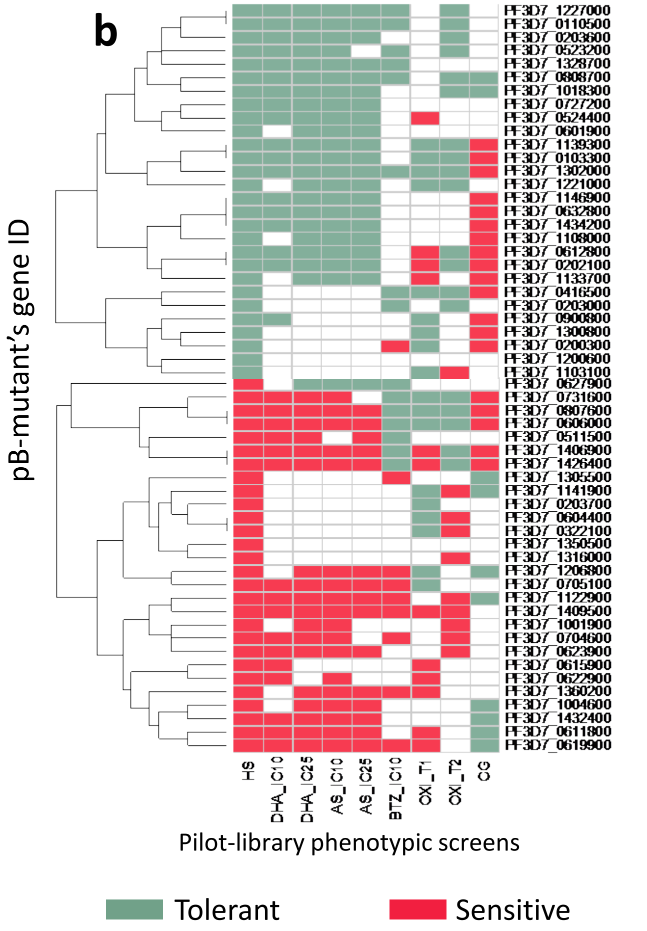

Supplementary Figure 2. Extended screening data against the pilot-library and summary.
a. Full drug-screening data for artemisinin-compounds AS and DHA, and proteasome-inhibitor Bortezomib (BTZ) against the pilot-library. HS-Sensitive mutants are significantly more sensitive to each drug than HS-Tolerant mutants. There is no significant relationship between *pB*-mutant sensitivity to any drug and mutant sensitivity in standard growth-conditions. (* two-tailed Wilcoxon $p < 0.05$; *** two-tailed Wilcoxon $p < 1e-10$).
b. HS-Sensitive (red) and HS-Tolerant (green) mutants and their phenotypes across all pooled phenotypic screens. Mutants are clustered by HS-phenotype. See Supplementary Data File 4.

Supplementary Figure 3.
Mutants in members of the DV proteome, targets of ART alkylation, and putative interacting partners of K13 tend to be sensitive to HS.

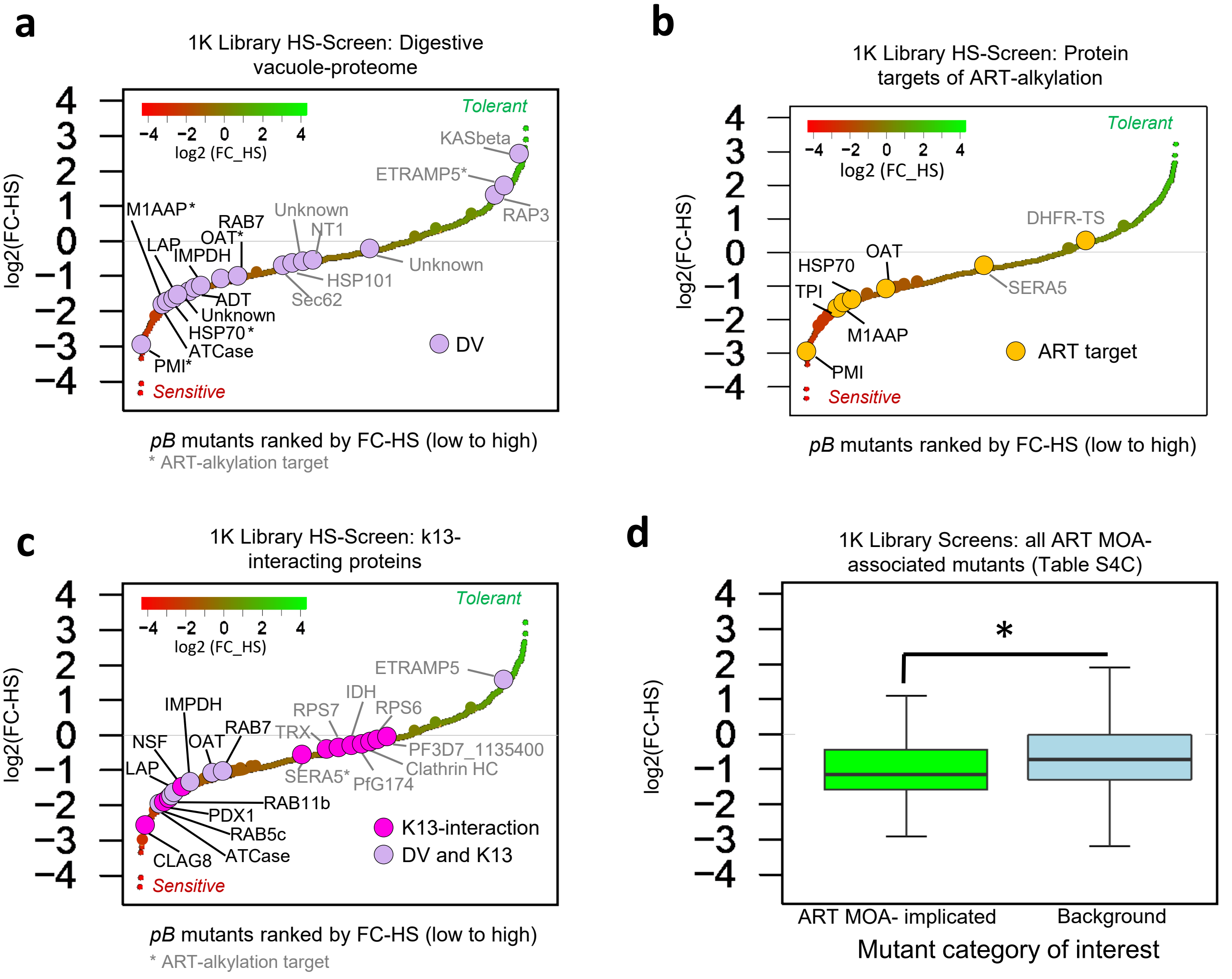

Supplementary Figure 3. Mutants in members of the DV proteome, targets of ART alkylation, and putative interacting partners of K13 tend to be sensitive to HS.
a. 1k HS-Screen mutants are ordered by FC-HS from HS-Sensitive to HS-Tolerant. Mutants in digestive vacuole-associated proteins as previously defined[1] are indicated in lavender dots. Gene-symbols for mutants with HS-sensitivity are labeled with black text (10 of 18 genes). Gene-symbols for HS-Neutral and HS-Tolerant mutants are labeled with grey text.
b. All mutants in ART alkylation-targets as previously defined[2] included in the 1K HS-Screen.
c. HS-screen phenotypes of mutants in putative K13-interacting proteins as previously defined[3].
d. Mutants in ART MOA-implicated processes are more sensitive to heat stress (but not normal growth conditions) than background. (n = 58 mutants in 47 unique genes total, vs. all mutants of the 1k library). * two-tailed Wilcoxon p-value = 0.03. Boxplots are drawn to present interquartile range of values (IQR). Lower bound of each box = 1st quartile, middle line = median, upper bound = 3rd quartile, and whiskers extend to at most 1.5x IQR. Source data are provided as a Source Data file.

Supplementary Figure 4.
Key processes are regulated in response to heat shock and artemisinin.

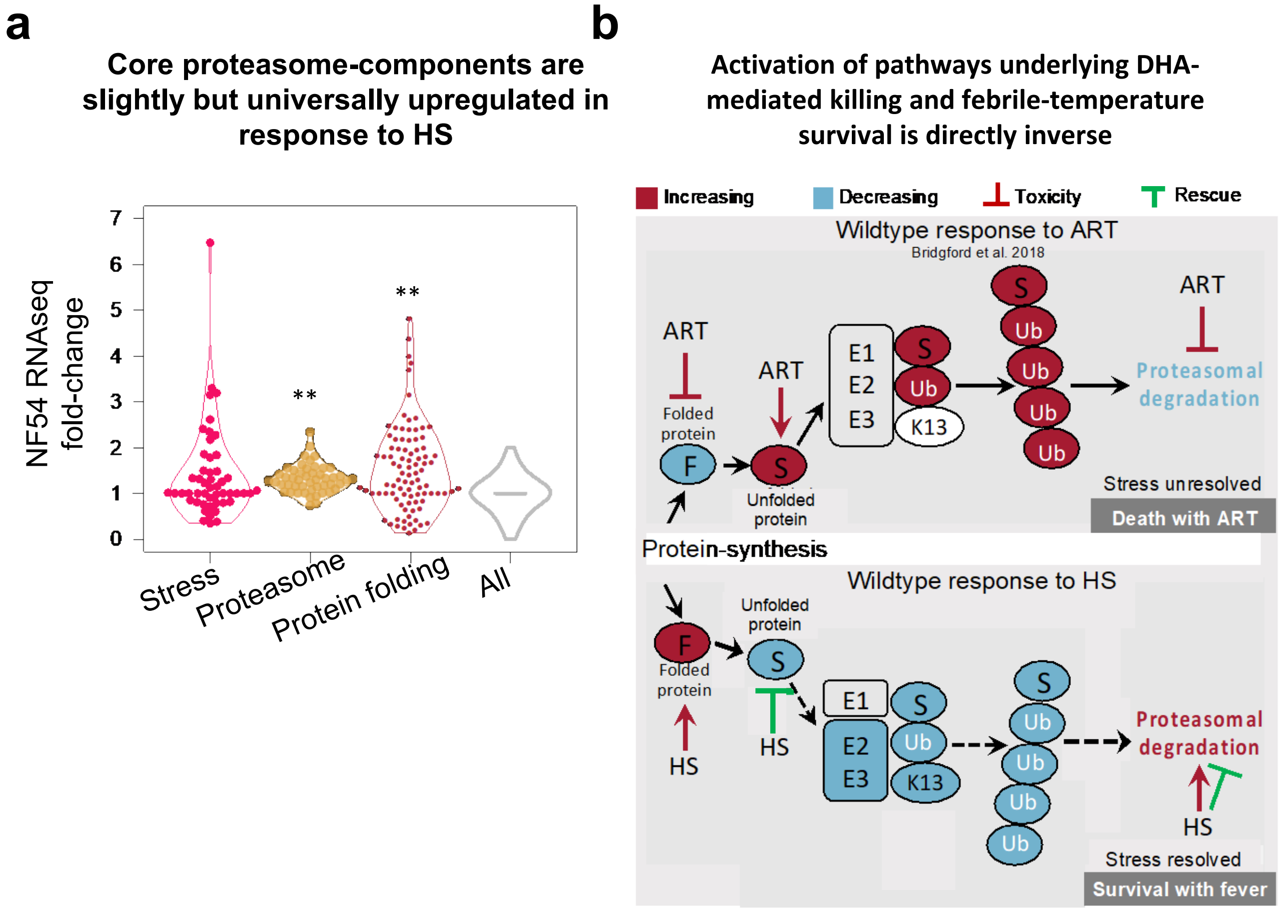

Supplementary Figure 4. Key processes are regulated in response to heat shock and artemisinin. a. Core proteasome-components are slightly but universally upregulated in response to HS as compared to other aggregately upregulated processes which have more heterogenous expression. Fold-change for most individual proteasome-components did not meet our threshold to be designated "upregulated". ** two-tailed Wilcoxon p-value < 1e-5.
b. Activation of pathways underlying DHA-mediated killing and febrile-temperature survival is directly inverse. Top. Model of DHA-mediated killing in *P. falciparum* adapted from [4]. Artemisinin (ART) damages and unfolds proteins, prevents folding of newly synthesized proteins, and inhibits the proteasome while at the same time activating E1/E2/E3 ubiquitin-machinery. Accumulation of toxic polyubiquitinated protein-substrates (S) overwhelms the cell and leads to death. **Bottom**. Model of parasite fever-response. Heat-stress causes globally damaged protein. The parasite increases the UPR as it inhibits E2/E3 ubiquitination to prevent accumulation of toxic, polyubiquitinated (Ub) protein-aggregates, while at the same time increasing its capacity for proteasome-mediated degradation—ultimately enabling the parasite to resolve heat-shock-instigated stress and survive febrile-temperatures.

Supplementary Figure 5.
QIseq data-correlations within and between pilot-library screens.

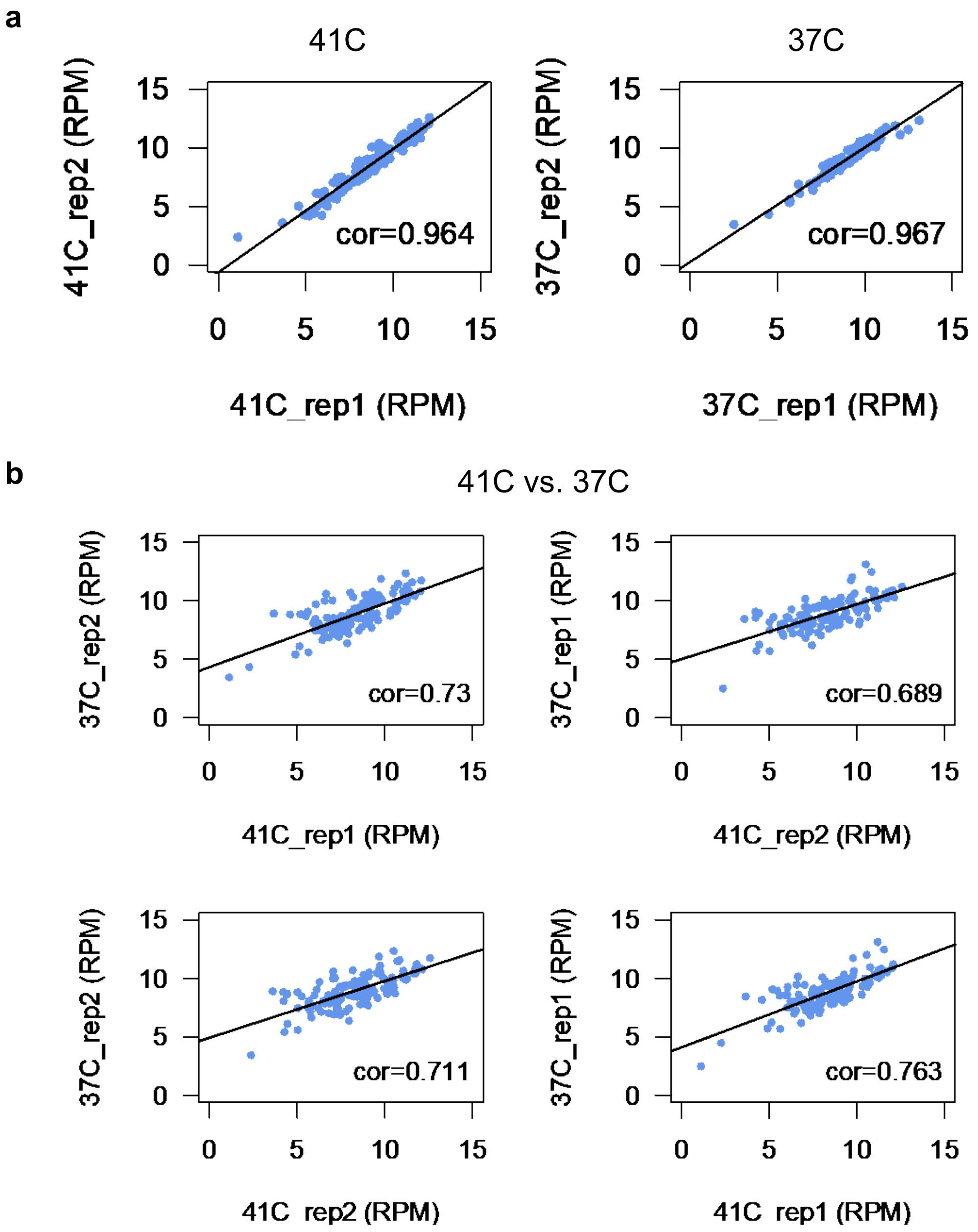

Supplementary Figure 5. QIseq data-correlations within and between pilot-library screens.
a. Pearson correlations between 5′ and 3′ QIseq data for 37°C_ideal-growth screen and 41°C_heat-shock screen indicate highly reproducible analyses across technical and biological replicates in both screens (Figure 1b).
b. Correlations within and between 37°C_ideal growth screen and 41°C_heat-shock screen QIseq data. The samples were collected in HS-Screens of the pilot-library include two bio-reps and three technical-reps (Figure 1a; Methods, HS-Screen). High correlations of two bio-reps within both HS-screens and Growth-screens (HS-Screen, R=0.94; Growth-Screen, R=0.89) indicate the pilot-library screens are highly reproducible, and weak correlation between HS-screens and Growth-screens (R = ~0.42) suggests heat-shock exposure-conditions were sufficient to allow reproducible detection of mutants with specific selection response-phenotypes from pooled screening.

Supplementary Figure 6.
***pB*-mutant insertion-site distribution in the pilot library and the 1K library.**

a

pB-mutants' distribution patterns in pilot-library and 1K-library

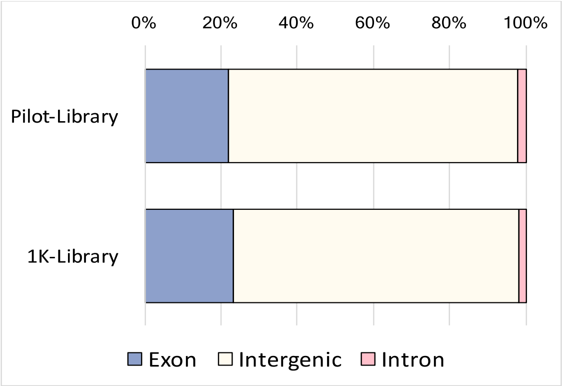

b

HS-Sensitive and HS-Tolerant pB-mutants' distribution patterns in 1K-library

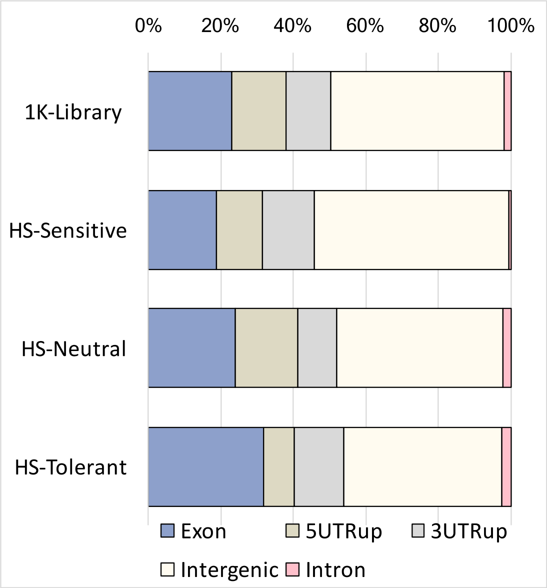

c

Distance between each pair of neighboring *piggyBac* insertions in 1K-library

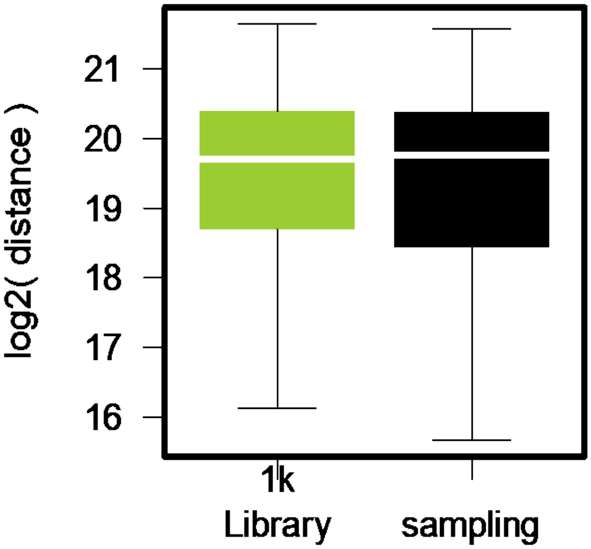

Supplementary Figure 6. *pB*-mutant insertions are randomly distributed in the pilot-library and the 1K-library.

a. Comparative analysis of the *piggyBac* mutants' distribution patterns in coding vs noncoding regions between the pilot-library and 1K-library. Distribution patterns between intergenic regions and CDS are almost equal, with composition also reflecting the distribution of the saturation mutagenesis-library as a whole[5]. See Supplementary Data Files 1-2.

b. *pB*-mutants' distribution patterns across HS phenotype-categories of the 1K library. HS-Tolerant mutants were more likely associated with dispensable genes (genes with exonic insertions) than HS-Sensitive genes.

c. Distances between insertions of the 1K library are random. There is no significant difference in distance between each pair of neighboring *piggyBac* insertions of the 1K library and coordinates chosen by random sampling (p-value = 0.787, two-tailed Mann-Whitney U test). Sampling was repeated 100x with sites randomly selected across all chromosomes. Boxplots are drawn to present interquartile range of values (IQR). Lower bound of each box = 1st quartile, middle line = median, upper bound = 3rd quartile, and whiskers extend to at most 1.5x IQR.

Supplementary Figure 7.
Reproducibility in the 1K-library HS-Screen.

a

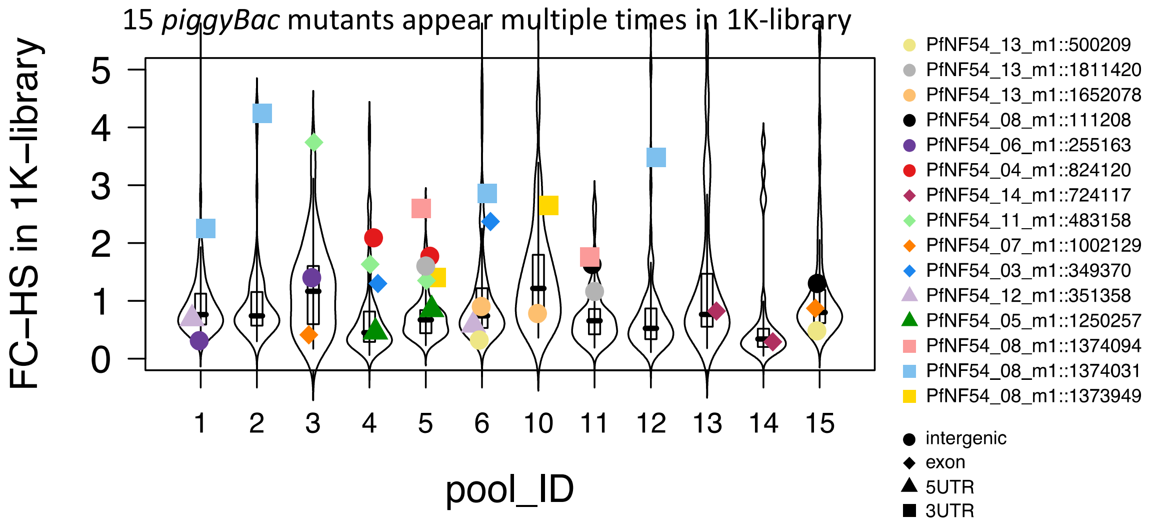

b

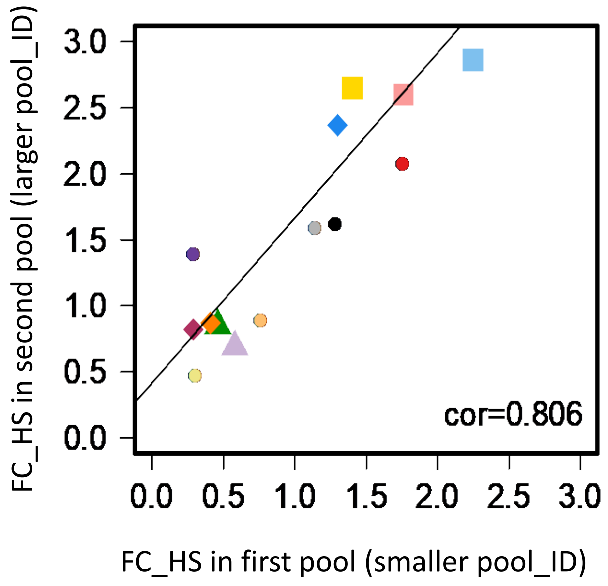

The Pearson correlation of 15 *piggyBac* mutants appear multiple times in 1K-library (cor = 0.806).

Supplementary Figure 7. Reproducibility of the 1K-library HS-Screen.
a. The 1K-library consists of randomly selected, uncloned large mixed-population pools (LMPP) of ~100 unique mutants per pool. Fifteen insertion-sites are duplicated in mutants of at least one other pool. Each of the 12 LMPP comprising the 1K-library (LMPP_1-6; LMPP_10-15) are indicated on the x-axis with violin plots showing the distribution of mutant fold change in response to heat shock (FC-HS) within that pool. FC-HS of the fifteen insertion-sites duplicated in at least one other pool are plotted in color, with insertion location-category indicated by shape. Boxplots are drawn to present interquartile range of values (IQR). Lower bound of each box = 1st quartile, middle line = median, upper bound = 3rd quartile, and whiskers extend to at most 1.5x IQR.
b. FC-HS of duplicated insertional mutants are highly correlated across pools (Pearson correlation = 0.806), indicating high reproducibility of mutant phenotypes independent of mutant pool-composition. Insertions are represented as in plot A.

Supplementary Figure 8.
Reproducibility within and between the pilot-library and 1K-library HS-Screens.

a

PB Pool ID	PB Mutant ID	Insertion Site	Distance to gene	Type of Insertion	GeneID	Gene Function	Fold Change (41C/37C)
Pilot-library	PB4_DHC_11	PfNF54_11_m1::879695	0	exon	PF3D7_1122900	dynein heavy chain, putative	0.21
1K-library-Pool_14	14-016_DHC_12B	PfNF54_12_m1::135712	0	exon	PF3D7_1202300	dynein heavy chain, putative	0.05
1K-library-Pool_4	4-054_DHC_10	PfNF54_10_m1::980010	0	exon	PF3D7_1023100	dynein heavy chain, putative	0.28
1K-library-Pool_14	14-015_DHC_12A	PfNF54_12_m1::121344	0	exon	PF3D7_1202300	dynein heavy chain, putative	0.32
Pilot-library	PB-54_FIKK9.3	PfNF54_09_m1::99320	0	exon	PF3D7_0902200	FIKK family (FIKK9.3)	0.52
1K-library-Pool_4	4-043_FIKK9.1	PfNF54_09_m1::92390	1095	intergenic	PF3D7_0902000	FIKK family (FIKK9.1)	0.23
1K-library-Pool_13	13-028_FIKK9.2	PfNF54_09_m1::93353	-477	3UTRup	PF3D7_0902100	FIKK family (FIKK9.2)	0.41
1K-library-Pool_12	12-040_ETRAMP	PfNF54_10_m1::82063	324	5UTRup	PF3D7_1001500	early transcribed membrane protein 10.1	0.310
1K-library-Pool_1	1-055_ETRAMP	PfNF54_10_m1::82163	424	5UTRup	PF3D7_1001500	early transcribed membrane protein 10.1	0.470
1K-library-Pool_4	4-048_HAD1	PfNF54_10_m1::1339529	-238	5UTRup	PF3D7_1033400	haloacid dehalogenase-like hydrolase (HAD1)	0.320
1K-library-Pool_12	12-037_HAD1	PfNF54_10_m1::1340834	201	3UTRup	PF3D7_1033400	haloacid dehalogenase-like hydrolase (HAD1)	0.410

b

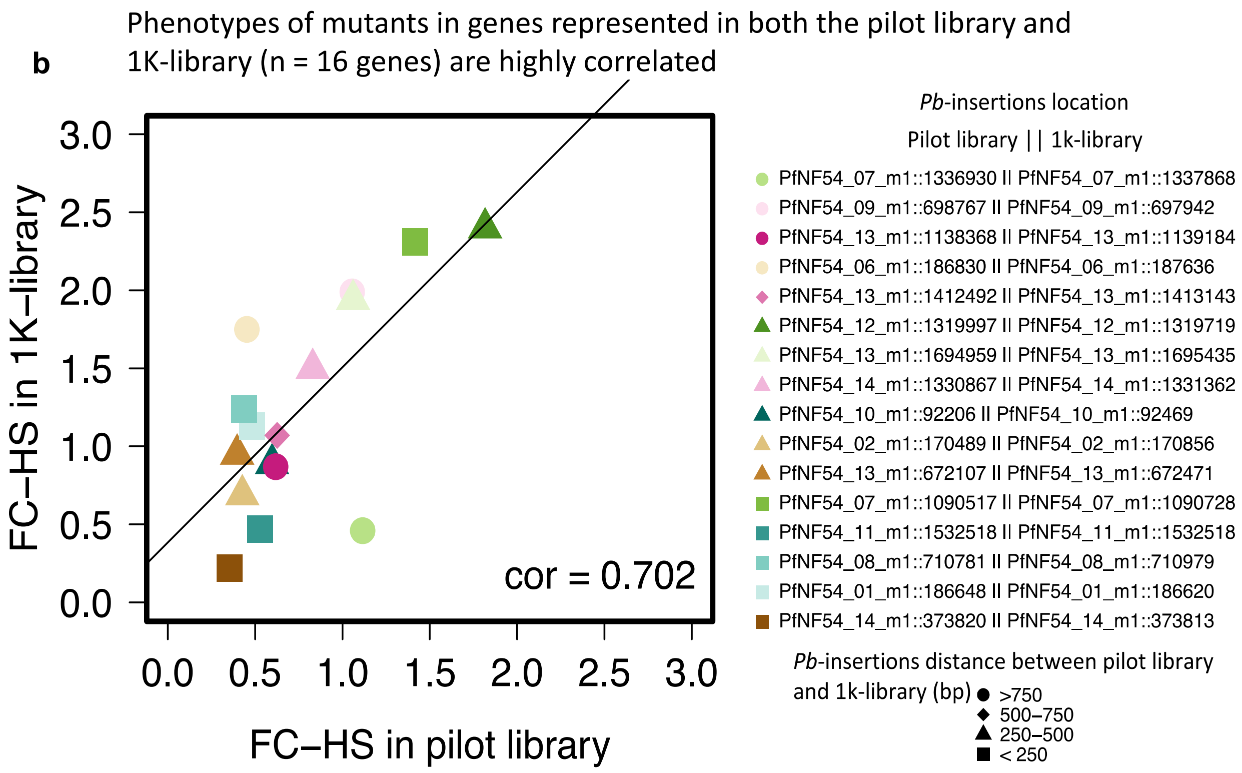

Supplementary Figure 8. Reproducibility within and between the pilot-library and 1K-library HS-Screens.

a. Within- and between-library consistency indicates the robusticity of HS phenotype-assignments in pooled screening. Dynein heavy chain (DHC) gene-family mutants (two in DHA_12, PF3D7_1202300; one mutant of DHA_10, PF3D7_1023100) were consistently identified as HS-Sensitive in the pilot-library and across multiple pools of the 1K-library, as were representatives of FIKK-family genes.

b. Heat shock phenotypes are reproducible between the pilot library and the 1K-library. FC-HS of insertional mutants in genes represented in both the pilot library and the 1K-library (n = 16 genes; colored points) are highly correlated (Pearson correlation = 0.702). Insertion coordinate in the pilot library is indicated on the left of the '||', while insertion coordinate of the mutant in the same gene in the 1K-library is to the right. Distance between the pilot-library insertion and the 1K-library insertion is indicated by shape (maximum distance = 1kb).

Supplementary Figure 9.
Distributions of PFS_{HS} for mutant heat-shock phenotype classifications in the 1K-Library screen.

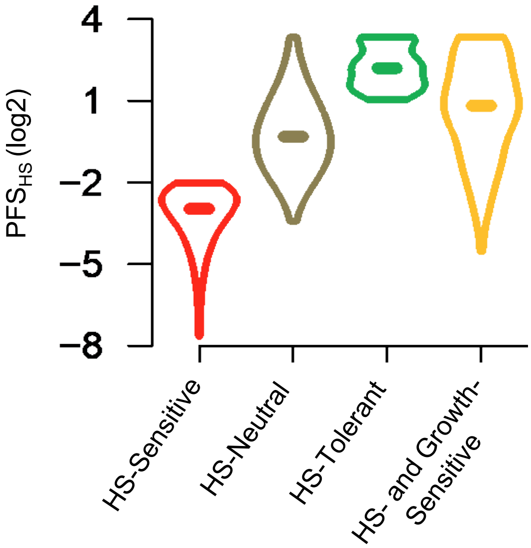

Supplementary Figure 9. Distributions of PFS_{HS} for mutant heat-shock phenotype classifications in the 1K-Library screen. HS-Sensitive mutants (mutants displaying defective growth in response to heat shock but not in response to ideal growth conditions) are assigned the lowest PFS_{HS}, while HS-Tolerant mutations are assigned the highest PFS_{HS}. See Supplementary Data File 2 and Methods for PFS_{HS} calculation details.

Supplementary Figure 10.
QIseq data-correlations between biological replicates for pilot-library phenotypic screens: drugs and oxidative stress.

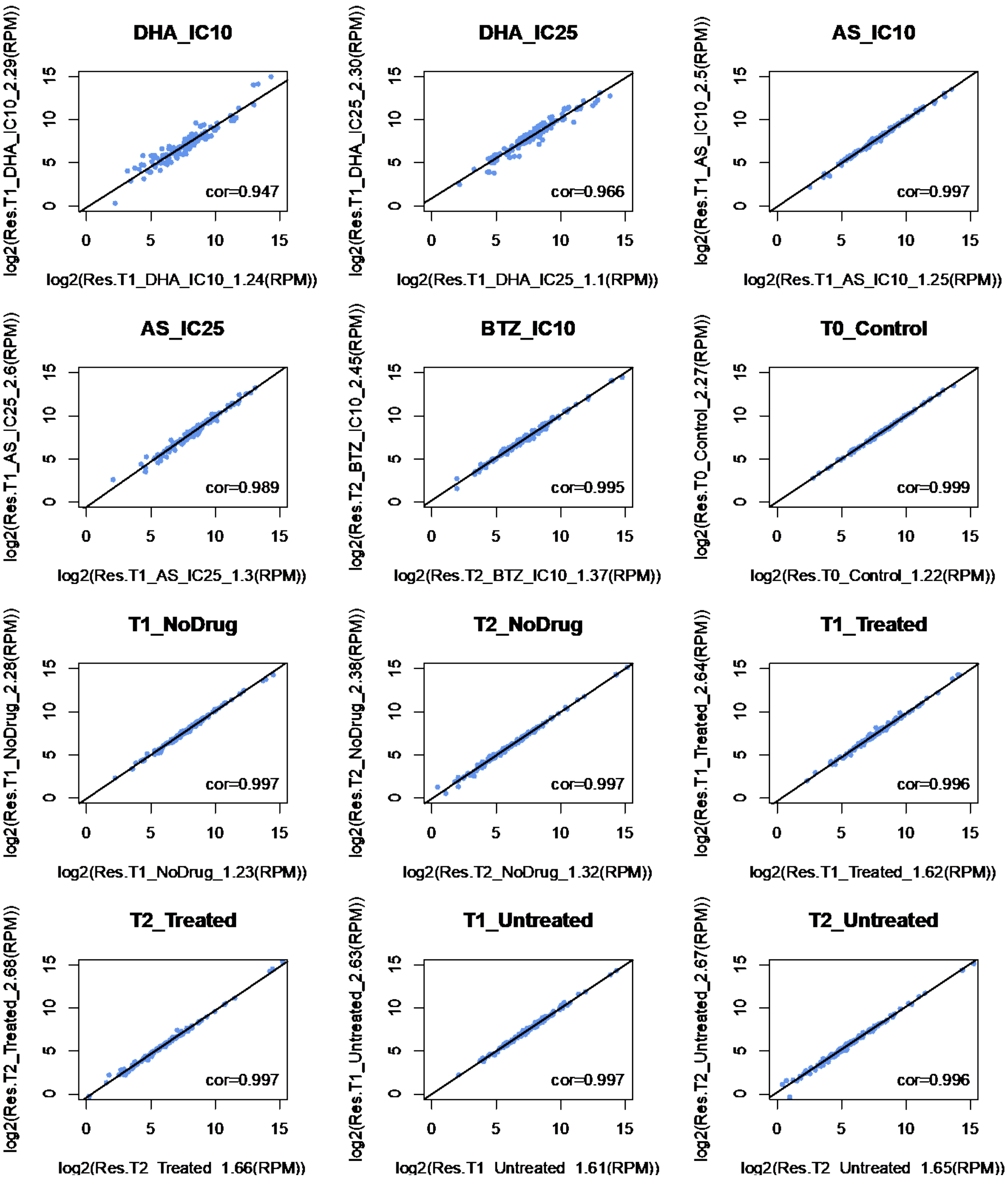

Supplementary Figure 10. QIseq data-correlations between biological replicates for pilot-library phenotypic screens: drugs and oxidative stress. All pooled phenotypic screens of the pilot-library (AS, DHA, BTZ, oxidative stress, ideal growth) were performed in biological duplicate. Biological replicates were highly correlated, indicating highly reproducible analyses across all pilot-library phenotype screens.

Supplementary Figure 11.
Methods and validation for comparative RNAseq.

RNA sample collection for wildtype malaria-parasite NF54 vs. two HS-Sensitive *pB*-mutant clones in response to febrile temperatures

a

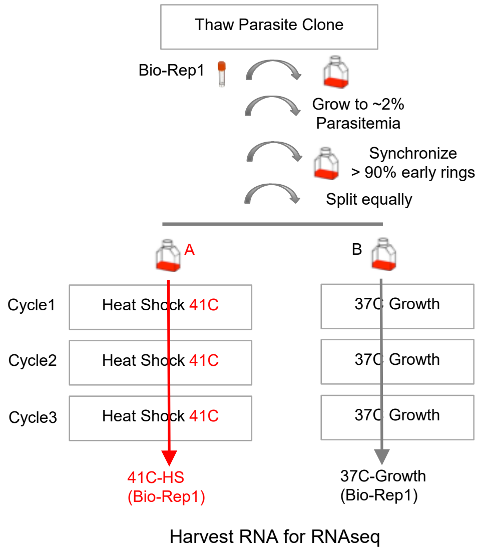

b

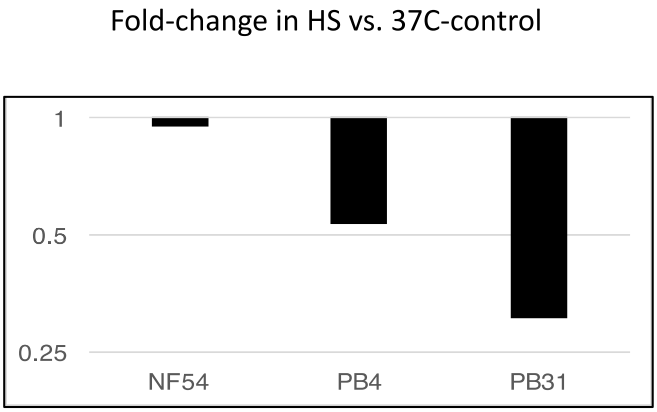

Supplementary Figure 11. Methods and validation for comparative RNAseq.
a. RNA sample-collection methods for wildtype malaria-parasite NF54 vs. two HS-Sensitive *pB*-mutant clones **Δ*DHC* (PB4) and Δ*LRR5* (PB31)** in response to febrile temperatures. Assays were performed in biological duplicate.
b. Validation of HS-Sensitive mutant-clones during RNA-Seq Sample preparation**.** Both mutants grown individually had growth-defects in response to HS as compared to NF54.

Supplementary Figure 12.
Complementary methods indicate genes driving the parasite heat-shock response.

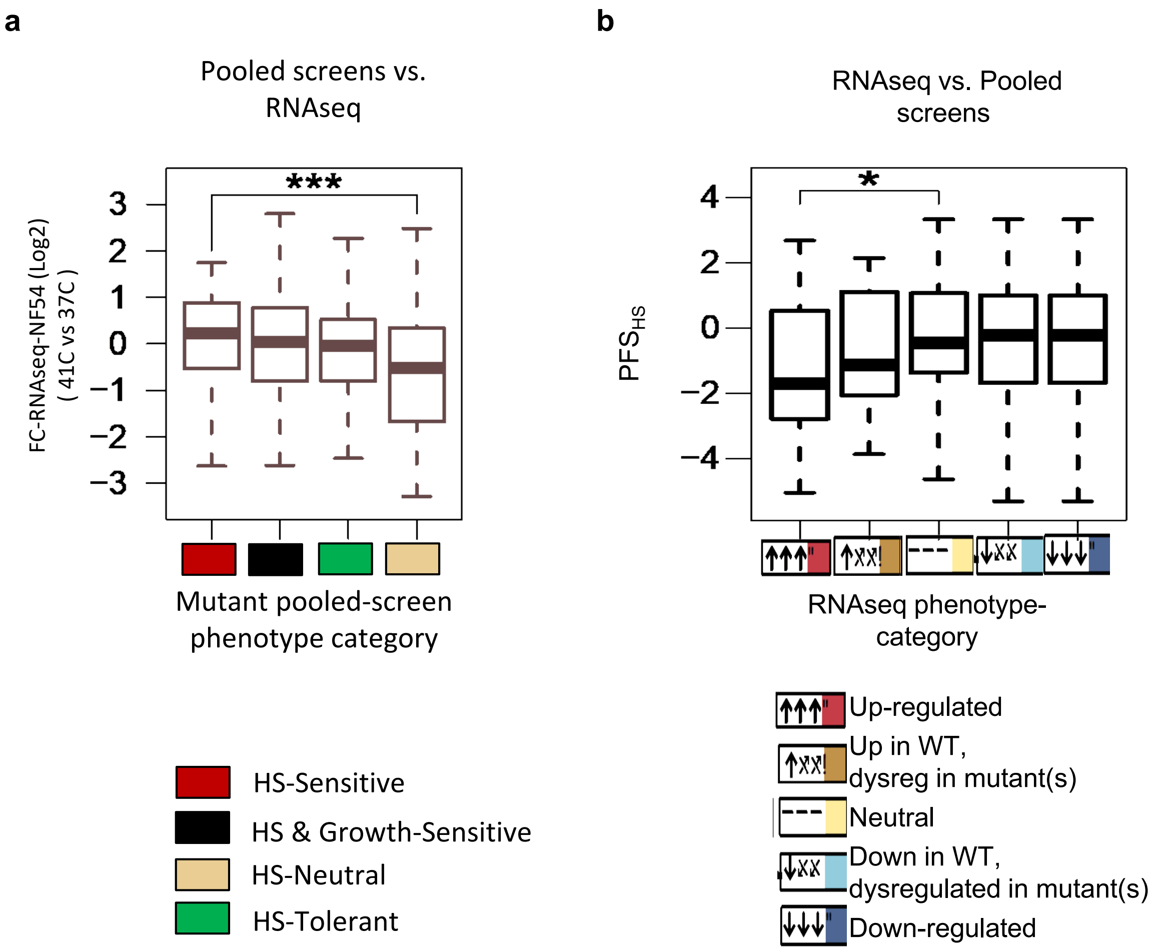

Supplementary Figure 12. Complementary methods (pooled phenotypic screening, phenotypic transcriptional profiling of HS-Sensitive mutants vs. wildtype in response to heat stress) indicate genes driving the parasite heat-shock response.

a. HS-Sensitive *pB* mutants tend to have mutations in genes that have significant changes in expression in response to heat-stress, while mutants that are neutral to or tolerant of heat-stress tend to have mutations in genes that are not regulated in response to heat-stress. (* two-tailed Wilcoxon $p < 0.05$; *** two-tailed Wilcoxon $p < 1e-10$). Boxplots are drawn to present interquartile range of values (IQR). Lower bound of each box = 1st quartile, middle line = median, upper bound = 3rd quartile, and whiskers extend to at most 1.5x IQR. See Supplementary Data File 2.

b. *pB* mutants in genes normally up-regulated in response to heat-stress grow poorly in response to heat-stress (i.e., have significantly lower phenotypic fitness-scores) than mutants in genes that are neutral or down-regulated in response to heat-stress. (* two-tailed Wilcoxon $p < 0.05$; *** two-tailed Wilcoxon $p < 1e-10$). Boxplots are drawn to present interquartile range of values (IQR). Lower bound of each box = 1st quartile, middle line = median, upper bound = 3rd quartile, and whiskers extend to at most 1.5x IQR. See Supplementary Data File 2.

SUPPLEMENTARY TABLE LEGENDS

Supplementary Table 1. QIseq dataset accession numbers.

Supplementary Table 1. Raw Qiseq data accession numbers.

Analysis provided in:	QIseq Sample Name	QIseq Sample ID	Accession number
SuppDataFile1	41C_A1_5'	HS-6-MP_A1	ERS571589
SuppDataFile1	41C_B1_5'	HS-7-MP_B1	ERS571592
SuppDataFile1	41C_C1_5'	HS-8-MP_C1	ERS571594
SuppDataFile1	37C_Ctrl1_5'	HS-10-MP_E1	ERS571599
SuppDataFile1	41C_A2_5'	HS-11-MP_A2	ERS571602
SuppDataFile1	41C_B2_5'	HS-12-MP_B2	ERS571612
SuppDataFile1	41C_C2_5'	HS-13-MP_C2	ERS571615
SuppDataFile1	37C_Ctrl2_5'	HS-14-MP_D2	ERS571617
SuppDataFile1	37C_Ctrl3_5'	HS-15-MP_E2	ERS571620
SuppDataFile1	41C_A1_3'	HS-6-MP_A1	ERS571589
SuppDataFile1	41C_B1_3'	HS-7-MP_B1	ERS571592
SuppDataFile1	41C_C1_3'	HS-8-MP_C1	ERS571594
SuppDataFile1	37C_Ctrl1_3'	HS-10-MP_E1	ERS571599
SuppDataFile1	41C_A2_3'	HS-11-MP_A2	ERS571602
SuppDataFile1	41C_B2_3'	HS-12-MP_B2	ERS571612
SuppDataFile1	41C_C2_3'	HS-13-MP_C2	ERS571615
SuppDataFile1	37C_Ctrl2_3'	HS-14-MP_D2	ERS571617
SuppDataFile1	37C_Ctrl3_3'	HS-15-MP_E2	ERS571620
SuppDataFile2	37C_T1_Ctrl	LMPP_Contl_1	ERS801326
SuppDataFile2	37C_T1_Ctrl	LMPP_Contl_2	ERS801327
SuppDataFile2	37C_T1_Ctrl	LMPP_Contl_3	ERS801328
SuppDataFile2	37C_T1_Ctrl	LMPP_Contl_4	ERS801329
SuppDataFile2	37C_T1_Ctrl	LMPP_Contl_5	ERS801330
SuppDataFile2	37C_T1_Ctrl	LMPP_Contl_6	ERS801331
SuppDataFile2	37C_T1_Ctrl	LMPP_Contl_10	ERS801335
SuppDataFile2	37C_T1_Ctrl	LMPP_Contl_11	ERS801336
SuppDataFile2	37C_T1_Ctrl	LMPP_Contl_12	ERS801337
SuppDataFile2	37C_T1_Ctrl	LMPP_Contl_13	ERS801338
SuppDataFile2	37C_T1_Ctrl	LMPP_Contl_14	ERS801339
SuppDataFile2	37C_T1_Ctrl	LMPP_Contl_15	ERS801340
SuppDataFile2	41C_HS_T1	LMPP_HS_1	ERS801342
SuppDataFile2	41C_HS_T1	LMPP_HS_2	ERS801343
SuppDataFile2	41C_HS_T1	LMPP_HS_3	ERS801344
SuppDataFile2	41C_HS_T1	LMPP_HS_4	ERS801345
SuppDataFile2	41C_HS_T1	LMPP_HS_5	ERS801346
SuppDataFile2	41C_HS_T1	LMPP_HS_6	ERS801347
SuppDataFile2	41C_HS_T1	LMPP_HS_10	ERS801351
SuppDataFile2	41C_HS_T1	LMPP_HS_11	ERS801352
SuppDataFile2	41C_HS_T1	LMPP_HS_12	ERS801353
SuppDataFile2	41C_HS_T1	LMPP_HS_13	ERS801354
SuppDataFile2	41C_HS_T1	LMPP_HS_14	ERS801355

SuppDataFile2	41C_HS_T1	LMPP_HS_15	ERS801356
SuppDataFile2	37C_T2_3Cycle growth	LMPP_Cycle6_1	ERS801358
SuppDataFile2	37C_T2_3Cycle growth	LMPP_Cycle6_2	ERS801359
SuppDataFile2	37C_T2_3Cycle growth	LMPP_Cycle6_3	ERS801360
SuppDataFile2	37C_T2_3Cycle growth	LMPP_Cycle6_4	ERS801361
SuppDataFile2	37C_T2_3Cycle growth	LMPP_Cycle6_5	ERS801362
SuppDataFile2	37C_T2_3Cycle growth	LMPP_Cycle6_6	ERS801363
SuppDataFile2	37C_T2_3Cycle growth	LMPP_Cycle6_10	ERS801366
SuppDataFile2	37C_T2_3Cycle growth	LMPP_Cycle6_11	ERS801367
SuppDataFile2	37C_T2_3Cycle growth	LMPP_Cycle6_12	ERS801368
SuppDataFile2	37C_T2_3Cycle growth	LMPP_Cycle6_13	ERS801369
SuppDataFile2	37C_T2_3Cycle growth	LMPP_Cycle6_14	ERS801370
SuppDataFile2	37C_T2_3Cycle growth	LMPP_Cycle6_15	ERS801371
SuppDataFile4	Control, T0, Biorep1	T0_BioRep_1	ERS3340779
SuppDataFile4	Control, T1, Biorep1	T1_No drug_1	ERS3340780
SuppDataFile4	DHA_IC10, Biorep1	T1_DHA_IC10_1	ERS3340781
SuppDataFile4	DHA_IC25, Biorep1	T1_DHA_IC25_1	ERS3340782
SuppDataFile4	AS_IC10, Biorep1	T1_AS_IC10_1	ERS3340784
SuppDataFile4	AS_IC25, Biorep1	T1_AS_IC25_1	ERS3340785
SuppDataFile4	Control, T0, Biorep2	T0_BioRep_2	ERS3340787
SuppDataFile4	Control, T1, Biorep2	T1_No drug_2	ERS3340788
SuppDataFile4	DHA_IC10, Biorep2	T1_DHA_IC10_2	ERS3340789
SuppDataFile4	DHA_IC25, Biorep2	T1_DHA_IC25_2	ERS3340790
SuppDataFile4	AS_IC10, Biorep2	T1_AS_IC10_2	ERS3340792
SuppDataFile4	AS_IC25, Biorep2	T1_AS_IC25_2	ERS3340793
SuppDataFile4	Control, T2, Biorep1	T2_No drug_1	ERS3340795
SuppDataFile4	BTZ_IC10, Biorep1	T2_BTZ_IC10_1	ERS3340802
SuppDataFile4	Control, T2, Biorep2	T2_No drug_2	ERS3340803
SuppDataFile4	BTZ_IC10, Biorep2	T2_BTZ_IC10_2	ERS3340810
SuppDataFile4	Oxi_T1 Unreated, biorep1	T1_Untreated_1	ERS3340829
SuppDataFile4	Oxi_T1 Treated, biorep1	T1_Treated_1	ERS3340830
SuppDataFile4	Oxi_T1 Unreated, biorep2	T1_Untreated_2	ERS3340831
SuppDataFile4	Oxi_T1 Treated, biorep2	T1_Treated_2	ERS3340832
SuppDataFile4	Oxi_T2 Untreated, biorep1	T2_Untreated_1	ERS3340833
SuppDataFile4	Oxi_T2 Treated, biorep1	T2_Treated_1	ERS3340834
SuppDataFile4	Oxi_T2 Untreated, biorep2	T2_Untreated_2	ERS3340835
SuppDataFile4	Oxi_T2 Treated, biorep2	T2_Treated_2	ERS3340852

SUPPLEMENTARY REFERENCES

1. Lamarque M*, et al.* Food vacuole proteome of the malarial parasite Plasmodium falciparum. *Proteomics Clin Appl* **2**, 1361-1374 (2008).

2. Ismail HM*, et al.* Artemisinin activity-based probes identify multiple molecular targets within the asexual stage of the malaria parasites Plasmodium falciparum 3D7. *Proceedings of the National Academy of Sciences* **113**, 2080-2085 (2016).

3. Gnädig NF*, et al.* Insights into the intracellular localization, protein associations and artemisinin resistance properties of Plasmodium falciparum K13. *PLoS Pathog* **16**, e1008482 (2020).

4. Bridgford JL*, et al.* Artemisinin kills malaria parasites by damaging proteins and inhibiting the proteasome. *Nature Communications* **9**, 3801 (2018).

5. Zhang M*, et al.* Uncovering the essential genes of the human malaria parasite Plasmodium falciparum by saturation mutagenesis. *Science* **360**, (2018).
