# Supplementary material for: The apicoplast link to fever-survival and artemisinin-resistance in the malaria parasite
Source: Nat Commun. 2021 Jul 27;12:4563. doi: 10.1038/s41467-021-24814-1 (PMC8316339; doi:10.1038/s41467-021-24814-1)
Supplement: Supplementary file 7 — Description of additional supplementary files [file 41467_2021_24814_MOESM7_ESM.docx]

Description of additional supplementary files

Title: Supplementary Data File 1.

Description: Pooled HS-Screen results of the P. falciparum pB-mutant pilot-library. A. Pooled HS-screen data supporting heat-shock phenotype assigned to each mutant of the pilot library (n = 128). B. Summary counts of pilot-library mutants by phenotype-category in pooled screening. C. GeneIDs, functional information, and distance to the insertion-site for neighboring genes on both sides of piggyBac insertions of the pilot library.

Title: Supplementary Data File 2.

Description: Pooled HS-Screen results of the 1K-library. A. Pooled HS-screen data supporting heat-shock phenotype assigned to each mutant of the 1K library (n = 922). B. Summary counts of 1K-library mutants by phenotype-category in pooled screening. C. Gene IDs, functional information, and distance to the insertion-site for neighboring genes on both sides of piggyBac insertions of the 1K-library.

Title: Supplementary Data File 3.

Description: Comparative RNAseq-results between NF54 and HS-Sensitive mutant-clones ΔLRR5 and ΔDHC in response to heatshock. A. All genes classified into HS response-categories in NF54 with or without exposure to heat-shock using RNAseq data (n = 2567). HS-classifications for each gene in two HS-Sensitive mutant-lines are indicated where available. Criteria for inclusion: NF54 expression above threshold (FPKM > or = 20 for at least one replicate in at least one temperature-condition) and FC-HS supported by two biological replicates. B. Genes included in functional enrichment-analyses. Criteria for inclusion: all genes with expression above threshold AND agreement between replicates as to HS fold-change classification for all three parasite lines (n = 1298). C. Full functional enrichment-results for all HS response-categories.

Title: Supplementary Data File 4.

Description: Drug- and oxidative stress-screen results of the pilot library. A. Final phenotype-assignments for each parallel screen of the pilot library. Screens: HS = heat shock. DHA = dihydroartemisinin. AS = artesunate. AM = artemether. BTZ = bortezomib. Screened IC-values of each drug are indicated after the underscore. OXI_T1, OXI_T2: oxidative stress at timepoints 1 and 2, respectively. A phenotype of “X” indicates mutants not meeting criteria to be classified as “Sensitive” or “Tolerant”. B. Fold-change and raw QIseq-data for drug and oxidative-stress screens of the pilot library.
