# Supplementary material for: The apicoplast link to fever-survival and artemisinin-resistance in the malaria parasite
Source: Nat Commun. 2021 Jul 27;12:4563. doi: 10.1038/s41467-021-24814-1 (PMC8316339; doi:10.1038/s41467-021-24814-1)
Supplement: Supplementary file 8 — Reporting Summary [file 41467_2021_24814_MOESM8_ESM.pdf]

nature research

Corresponding author(s): John H Adams, NCOMMS-20-21405C

Last updated by author(s): Jun 10, 2021

# Reporting Summary

Nature Research wishes to improve the reproducibility of the work that we publish. This form provides structure for consistency and transparency in reporting. For further information on Nature Research policies, see our Editorial Policies and the Editorial Policy Checklist.

## Statistics

For all statistical analyses, confirm that the following items are present in the figure legend, table legend, main text, or Methods section.

| n/a | Confirmed | |
|---|---|---|
| ☐ | ☒ | The exact sample size (*n*) for each experimental group/condition, given as a discrete number and unit of measurement |
| ☐ | ☒ | A statement on whether measurements were taken from distinct samples or whether the same sample was measured repeatedly |
| ☐ | ☒ | The statistical test(s) used AND whether they are one- or two-sided<br>*Only common tests should be described solely by name; describe more complex techniques in the Methods section.* |
| ☐ | ☒ | A description of all covariates tested |
| ☐ | ☒ | A description of any assumptions or corrections, such as tests of normality and adjustment for multiple comparisons |
| ☐ | ☒ | A full description of the statistical parameters including central tendency (e.g. means) or other basic estimates (e.g. regression coefficient) AND variation (e.g. standard deviation) or associated estimates of uncertainty (e.g. confidence intervals) |
| ☐ | ☒ | For null hypothesis testing, the test statistic (e.g. *F*, *t*, *r*) with confidence intervals, effect sizes, degrees of freedom and *P* value noted<br>*Give P values as exact values whenever suitable.* |
| ☐ | ☒ | For Bayesian analysis, information on the choice of priors and Markov chain Monte Carlo settings |
| ☐ | ☒ | For hierarchical and complex designs, identification of the appropriate level for tests and full reporting of outcomes |
| ☐ | ☒ | Estimates of effect sizes (e.g. Cohen's *d*, Pearson's *r*), indicating how they were calculated |

*Our web collection on statistics for biologists contains articles on many of the points above.*

## Software and code

Policy information about availability of computer code

| | |
|---|---|
| Data collection | No software was used for the data collection of this study. |
| Data analysis | TopHat 2.1.1Cufflink 2.2.1, R3.4, R library 'Mixtools 1.1.0', GGplot2 3.2.1, Gplots 3.0.1 |

For manuscripts utilizing custom algorithms or software that are central to the research but not yet described in published literature, software must be made available to editors and reviewers. We strongly encourage code deposition in a community repository (e.g. GitHub). See the Nature Research guidelines for submitting code & software for further information.

## Data

Policy information about availability of data

All manuscripts must include a data availability statement. This statement should provide the following information, where applicable:

- Accession codes, unique identifiers, or web links for publicly available datasets
- A list of figures that have associated raw data
- A description of any restrictions on data availability

All data and code to understand and assess the conclusions of this research are available in the main text and supplementary materials and are deposited into the European Nucleotide Archive (ENA; http://www.ebi.ac.uk/ena/). Accession numbers for individual experiments and libraries are listed in Table S7.

# Field-specific reporting

Please select the one below that is the best fit for your research. If you are not sure, read the appropriate sections before making your selection.

☒ Life sciences ☐ Behavioural & social sciences ☐ Ecological, evolutionary & environmental sciences

For a reference copy of the document with all sections, see nature.com/documents/nr-reporting-summary-flat.pdf

# Life sciences study design

All studies must disclose on these points even when the disclosure is negative.

| | |
|---|---|
| Sample size | We first used a pool of 128 unique, extensively characterized P. falciparum pB-mutant clones reflecting disruptions in genes spanning a range of functional categories, as well as many genes without existing functional information, as a “pilot library” for initial phenotypic screen-development (see Methods). We next scaled our pooled HS-screen to the 1K-library comprised 922 functionally uncharacterized mutants randomly selected from our saturation library using the methods we established in our pilot-library screens. |
| Data exclusions | QIseq Data: the reads numbers were removed for data analysis if the sequence not containing the correct QIseq integration sequence (TAGGGTTAANNN for both 5' and 3' libraries, NNN is genomic sequence after the TTAA integration site) or not mapped against the P. falciparum NF54 genome.<br>RNAseq Data: the genes in RNA_Seq were removed for downstream analysis if the FPKM value is lower than 20. |
| Replication | All experiments were reproduced to reliably support the conclusions stated in the manuscript. |
| Randomization | We previously used random piggyBac-transposon insertional mutagenesis to uncover genes essential for P. falciparum blood-stage survival, generating a saturation-level P. falciparum mutant library containing ~38,000 single-disruption mutants (Zhang et al. 2018). In this study, we present the first large-scale forward-genetic functional screen in P. falciparum to identify factors linked to parasite survival of febrile temperatures using the 1K-library comprised mutant-pools randomly selected from our saturation library, covering genes annotated to diverse GO-categories, as well as many genes of unknown function. |
| Blinding | QIseq-data resulting from the pooled HS- and Growth-screens allowed robust assignment of mutant-phenotypes for both (see Methods). QIseq identified the samples and mutants by the unique sequencing index and transposon insertion site. |

# Reporting for specific materials, systems and methods

We require information from authors about some types of materials, experimental systems and methods used in many studies. Here, indicate whether each material, system or method listed is relevant to your study. If you are not sure if a list item applies to your research, read the appropriate section before selecting a response.

## Materials & experimental systems

| n/a | Involved in the study | |
|---|---|---|
| ☒ | ☐ | Antibodies |
| ☐ | ☒ | Eukaryotic cell lines |
| ☒ | ☐ | Palaeontology and archaeology |
| ☒ | ☐ | Animals and other organisms |
| ☒ | ☐ | Human research participants |
| ☒ | ☐ | Clinical data |
| ☒ | ☐ | Dual use research of concern |

## Methods

| n/a | Involved in the study | |
|---|---|---|
| ☒ | ☐ | ChIP-seq |
| ☒ | ☐ | Flow cytometry |
| ☒ | ☐ | MRI-based neuroimaging |

## Eukaryotic cell lines

Policy information about cell lines

| | |
|---|---|
| Cell line source(s) | Human malaria parasite Plasmodium falciparum were generated on the NF54 clone background as described previously (Zhang et al. 2018). |
| Authentication | Plasmodium falciparum NF54 authenticated by WGS method at Sanger UK as described previously (Zhang et al. 2018). |
| Mycoplasma contamination | All Plasmodium falciparum NF54 mutant clones were tested for mycoplasma contamination. |
| Commonly misidentified lines<br>(See ICLAC register) | None of the used cell lines is listed in ICLAC database. |
